# Impact of elicitors and light on biosynthesis of sesquiterpene lactones in tissue culture of *Arnica montana* and its variety Arbo

**DOI:** 10.3389/fpls.2025.1611849

**Published:** 2025-06-19

**Authors:** Agata Parafiniuk, Krystyna Kromer, Mariusz G. Fleszar, Katarzyna Wróblewska, Jerzy Ł. Wiśniewski, Andrzej Gamian

**Affiliations:** ^1^ Plant Tissue Culture Laboratory, Botanical Garden, Faculty of Biological Sciences, University of Wroclaw, Wroclaw, Poland; ^2^ Department of Biochemistry and Immunochemistry, Wroclaw Medical University, Wroclaw, Poland; ^3^ Omics Research Center, Wroclaw Medical University, Wroclaw, Poland; ^4^ Department of Horticulture, Wroclaw University of Environmental and Life Sciences, Wrocław, Poland; ^5^ Department of Biochemistry, Molecular Biology and Biotechnology, Faculty of Chemistry, Wroclaw University of Science and Technology, Wroclaw, Poland; ^6^ Hirszfeld Institute of Immunology and Experimental Therapy, Polish Academy of Sciences, Wroclaw, Poland

**Keywords:** sesquiterpene lactones biosynthesis, helenalin, dihydrohelenalin, *Arnica montana*, *in vitro* culture, elicitors, bioreactor

## Abstract

*Arnica montana* is a popular medicinal plant known for the sesquiterpene lactones (SLs) found in its flowers, which are valuable components in pharmacology and cosmetology. We present the application of *in vitro* culture of the species *A. montana*, representing the dihydrohelenalin type and Arbo variety, richer in helenalin, as an alternative to the flower plantations method for acquiring these metabolites. We specify culture conditions under which the SL content was determined using high-resolution liquid chromatography with quadrupole time-of-flight mass spectrometry (LC-QTOF-MS). On a basic Murashige and Skoog (MS) medium without growth regulators, an increase in light intensity within the 50–150 µmol m^-2^ s^-1^ positively stimulated SLs synthesis. A high content of SLs was confirmed in *in vitro* leaves of both species, particularly at the maximum tested light intensity of 150 µmol µmol m^-2^ s^-1^ (*A. montana*: 32.57 mg/g DW; Arbo variety: 73.90 mg/g DW). Chitosan, jasmonic acid (JA), and red light alter the spectrum of the present SLs, favoring helenalin derivatives. The study contributes to understanding light's intensity and spectrum in Arnica's leaf development process. It confirms the pivotal role of phytochrome in inducing the expression of GA biosynthetic genes that control trichome emergence and stimulate sesquiterpene lactones formation.

## 1 Introduction

Natural treatment approaches have grown in popularity in recent years. The increasing sales of herbal medicines, nutraceuticals, and cosmetics with natural ingredients have raised the demand for plant raw materials from natural sources. *Arnica*, a member of the Asteraceae family, is an ingredient in many cosmetic and pharmaceutical products (Alamgir, 2018). It has anti-inflammatory, analgesic, and anti-swelling properties in external applications. Furthermore, it exhibits antibacterial activity, especially against Gram-positive bacteria, and possesses antifungal and antioxidant properties (Kriplani et al., 2017). It also enhances blood supply, aids in hematoma resorption, and accelerates granulation (Šutovská et al., 2014; Kriplani et al., 2017; Petrova et al., 2024).

The main active compounds in *A. montana* are the SLs, such as helenalin, dihydrohelenalin, and their esters with carboxylic acids. At the end of the 20^th^ century, Rüngeler et al. (1999) discovered that helenalin and its derivatives inhibit the nuclear factor kappa-light-chain-enhancer of activated B cells (NFκB). This factor regulates the expression of pro-inflammatory genes involved in immune processes by covalently binding to a cysteine residue (C38) within the DNA interface of the 65 kDa (p65) subunit of the transcription factor, thereby blocking the ability of this factor to attach to DNA (Lyss et al., 1997, 1998; Rüngeler et al., 1999; Schmidt et al., 1999; Garcia-Pineres et al., 2001). Comparative tests have demonstrated that helenalin inhibits the transcription factor twice as effectively as dihydrohelenalin (Klaas et al., 2002).

*Arnica* is known for its significant medicinal properties, which have led to its harvesting from the wild in large quantities, and this practice causes the depletion of its plant populations. Since the late 1990s, the trade in arnica flowers and roots has been monitored in the European Union. The plants are primarily collected from natural habitats, and the pharmacopeial raw materials used are the inflorescences (*Anthodium arnicae*) (European Directorate for the Quality of Medicines & HealthCare (EDQM), 2022). According to Lange (1998), the European market utilizes around 50,000 kg of dry flowers yearly. Requirements substantially exceed the existing supply, and cosmetic companies are interested in any amount of raw material; additionally, demand for this raw material is predicted to increase further by 2030 (Fact M. R., 2022).

To address this shortage, herbal companies, such as Bauer, have initiated efforts to develop new methods for cultivation. These methods focus on breeding varieties characterized by greater vigor than ecotypes that naturally appear in the environment (Smallfield and Douglas, 2008). However, in France, the Sicarappam company encourages producers to experimentally cultivate wild forms of this species that ensure that biodiversity is preserved under the standards of organic farming and grows *A. montana* crop near its natural habitat (Sicarappam, 2024). In Poland, a team from the University of Life Sciences in Lublin is working on the cultivation of the wild species *A. montana*, analyzing methods of fertilization, reproduction, and the value of obtained flower extracts (Sugier et al., 2013, 2017). More research employing modern biotechnology is required because of the growing demand for raw resources and the need to protect *A. montana* in its natural habitat.

The pioneering work on the analysis of the content of SLs in the *in vitro* culture of *A. montana* was conducted by Malarz et al. (1993), who investigated the effect of cytokinins 6-(γ,γ-dimethylallylamino) purine (2iP), 6-benzylaminopurine (BAP), kinetin (Kn), and auxins 1-naphthaleneacetic acid (NAA) and 2,4-dichlorophenoxyacetic acid (2,4-D) on shoot proliferation. The authors identified the optimal medium containing NAA (0.5 mg/L) and Kn (2.5 mg/L). In the analyzed culture samples, the helenalin acetate (HA) in plant leaves was estimated at 0.073%, while in the leaves of proliferating shoots, it was 0.016% of dry weight (DW). As assumed by Malarz et al. (1993), the presence of HA may depend on the developmental stage and the presence of roots.Weremczuk-Jeżyna and Wysokińska (2000; 2004) and Pawłowska et al. (2005) studied the possibilities of using various types of *A. montana* cultures, including callus tissue, cell suspensions, shoots, and transgenic roots in the production of secondary metabolites. Phytochemical analyses showed that both transgenic roots and arnica plants obtained from cultures produced SLs (helenalin, dihydrohelenalin, dihydrohelenalin acetate), sterols (sitosterol, stigmasterol), phenolic acids (chlorogenic acid, caffeic acid), thymol derivatives, and essential oil. However, because of the small quantity of these metabolites produced in culture, the path to exploiting this plant in biotechnology-based manufacturing processes remains long (Weremczuk-Jeżyna and Wysokińska, 2000, 2004; Pawłowska et al., 2005).

Stefanache et al. (2014) found that the SL content in *A. montana* plants from *in vitro* culture reached 1.29%, comparable to that found in superelite flowers from field cultivation. Petrova et al. (2014) demonstrated the *in vitro* propagation of *A. montana* using both liquid and solid agar media supplemented with the hormones BA and IAA. The total SL constituents determined by gas chromatography showed a level between 7.62–15.34 mg/g DW depending on the culture type (Petrova et al., 2014). The proposed culture technique in this study differs from the method described above in two respects, primarily the lack of plant hormones in the medium and culture illumination. These factors are probably responsible for the significantly lower content of SLs in the previously mentioned works. Additionally, we analyzed quantitatively the level of SLs of *A. montana* and the Arbo variety for the first time in *in vitro* conditions and field-grown plants with the same genome.

In plant cultures carried out for the biosynthesis of secondary metabolites, several manipulations on the composition of macronutrients are used, such as nitrogen, phosphorus, potassium, hormone content, and the addition of biotic elicitors imitate the attack of microorganisms, insects, and abiotic factors. The introduction of biotic elicitors such as chitosan or chitin into the substrate allows determining whether the biosynthesis of SLs is related to the response to stress caused by the attack of a wide range of fungi, leading to an increase in the expression of genes encoding defense proteins, including chitinases (Kisiel and Jęckowska, 2019). The substrate for these enzymes consists of N-acetylglucosamine and arabinogalactan residues from cell walls, which, through chitin oligosaccharide molecules, participate in signaling pathways that generate defense reactions. Plant chitinases of various classes and their decomposition products are finding increasingly wider industrial applications (Showalter, 2001).

As the literature review shows, advancements in *in vitro* technology have made it possible to increase the SL content in cultivated plants, achieving levels comparable to those found in field-grown plants. This raises the question of whether it is possible to identify the key factors determining the ability to biosynthesize lactones in sterile cultures, and whether this method holds application value. Some plant hormones, i.e., jasmonic acid (JA), are rapidly produced in response to herbivore attacks (Kessler and Baldwin, 2002). Some plant hormones, such as jasmonic acid (JA), regulate growth, secondary metabolism, and defense reaction against biotic and abiotic stresses, and produced rapidly in response to herbivore attacks (Kessler and Baldwin, 2002). JA is known to activate terpene production, influence terpene profiles, and increase the production of both volatile monoterpenes and sesquiterpenes in the leaves and fruits of kiwi, *Actinidia* (Wang et al., 2015). The various regulatory functions of this hormone are performed through CORONATINE INSENSITIVE 1 (COI1) receptor and through JASMONATE ZIM DOMAIN (JAZ) transcription repressor in plants that inhibits JA signaling downstream by TFs (Zhao et al., 2024). JAs influence growth-defense reactions and carbohydrate metabolism, alter metabolism of the tricarboxylic acid cycle, and regulate antioxidant and energy metabolism (Savchenko et al., 2019).

Similarly, the illumination intensity and light spectrum in the *in vitro* cultures enhance or inhibit the synthesis of specific groups of secondary metabolites and, in this respect, are important factors that can efficiently elicit the production of therapeutic compounds (Hashim et al., 2021). Specific electromagnetic wavelengths in the visible range of 380–700 nm, ultraviolet, and infrared can trigger biochemical variability of synthesized groups of secondary metabolites. The red spectrum of light induces the production of alkaloids, the blue spectrum of phenylpropanoids and flavonoids (Zhang et al., 2021), and the ultraviolet and blue spectrum of terpenes, although various unexpected plant reactions have been reported (Kim et al., 2007; Hashim et al., 2021).

Frey et al. (2024) published a summary review of SLs, which provides insight into their biosynthesis, regulation, and signaling roles. The biosynthesis of SLs has been described for several plant species within the Asteraceae, where 30 types of backbone SLs structures have been reported (Frey et al., 2024). Different types of sesquiterpene synthase are responsible for the formation of the primary 15-carbon ring by cyclization of farnesyl pyrophosphate (FPP) with a germacranolide, elemanolide, cadinanolide, and guaianolide skeleton by sesquiterpene synthases (Seaman, 1982; Lange and Lee, 1987), but one and the same synthase can form different products, because terpenoid synthases of secondary metabolism are often represented by multi-copy gene families (Bouwmeester et al., 2002; De Bruyn et al., 2023). Most SLs known to be biosynthesized are derived from the germacrene A structure, produced by the enzyme germacrene A synthase (GAS). In the next stage, germacrene A is oxidized to alcohol, aldehyde, and acid by germacrene A oxidase (GAO, CYP71A2-8) (Ikezawa et al., 2011; De Bruyn et al., 2023), and the formed germacrene A acid is an important intermediate for biosynthesis of other SLs.

A similar chain of reactions involving amorphadiene synthase, amorphadiene oxidase, and artemisinic aldehyde synthase was discovered in *Artemisia annua*. Subsequently, the double-bond reductase converts the AMO intermediate to dihydro-artemisinic aldehyde and to dihydro-artemisinic acid, the last enzymatic step to the SL artemisinin. Nonenzymatic photoreaction in trichomes is responsible for transforming dihydro-artemisinic acid to lactone (Czechowski et al., 2016).

In the case of *A. montana*, the synthesized helenaloids belong to the pseudoguainolides type. In theoretical studies, Barquera-Lozada and Cuevas (2009) confirmed the hypothetical mechanisms of Hendrickson and Fischer of the biogenetic origin of pseudoguaianolide lactones by transformation from germacranolide, via guainolide, to pseudoguainolide obtained experimentally by Ortega and Maldonado (1989) in the presence of bentonitic earth. It was also described as electrophilic intramolecular cyclizations or acid-mediated rearrangements of 15-hydroxygermacranolides, salonitenolide, and artemisiifolin to the lactones with different skeletons of the eudesmanolides, guainolides, amorphanolides, or other germacranolides (Álvarez-Calero et al., 2018). CYPs, converted enzymes from the CYP71BL subfamily, perform hydroxylation of germacrene A acid; they are also responsible for further transformations and rearrangements of the sesquiterpene backbone structure of germacrene acid (Frey et al., 2018), costunolides. In the helenalin structure, the most important functional groups are the α-methylene and cyclopentanone ring. Hydroxyl groups on the backbones can be esterified to aliphatic and aromatic organic acids.

The main genes responsible for terpenoid biosynthesis in *Arabidopsis* are terpene synthases (TPS), AtTPS11 and AtTPS21. In *Freesia x hybrida*, the biosynthesis and emission of monoterpenes are mainly controlled by the TF AtMYC2, cooperating with the factor AtMYB21 (Yang et al., 2020). In roses, up-regulation of terpenoid level stimulates overexpression of PAP1, and in *Artemisia annua* by ERF and AaWRKY9 TFs (Zvi et al., 2012). The basic helix-loop-helix bHLH and MYB transcription factors regulate the transduction of signal in linalool synthesis (Yang et al., 2024), and light signals interact with photoreceptors, controlling phytochrome. In *Arabidopsis*, phB binds to PIF4 and PIF5 (PHYTOCHROME INTERACTING FACTOR), promoting their ubiquitination and mediating positive regulation of anthocyanin accumulation (Jeong and Choi, 2013). Similarly, the response to PIF3 was noticed but requires HY5 (Kim et al., 2003; Liu, 2013) for action. The phyB and phyB genes regulate the expression of chlorophyll accumulation, interacting with PIF1 (Huq et al., 2004; Leivar and Monte, 2014). PIFs are also involved in CRY1-mediated blue light signaling for secondary metabolite biosynthesis. Moreover, PIF4 and CRY1 form a complex that suppresses blue light transcriptional activity, repressing auxin synthesis (Ma et al., 2016; Zhou et al., 2019), whereas Han et al. (2022) showed that AmPIF4 contributes to blue light-induced terpenoid biosynthesis.

This study aimed to determine the impact of several known elicitors on the profile of SLs produced in *A. montana* and Arbo variety plant cultures *in vitro*, to define the likely sites of biosynthesis of these therapeutic substances, and to assess the usefulness of this method as an alternative source for protecting the species’ natural resources.

## 2 Materials and methods

### 2.1 Plant material and initiation of culture

The culture was initiated from selected seeds of *Arnica montana* from the Hala Izerska site at an altitude of 840–880 m n.p.m., Sudety Mountain, Poland, and Arbo variety seeds from a private planter from Germany. The seeds were placed on filter paper (Whatman, Grade 1, 11 µm) and immersed in 70% ethanol (Merck Millipore, Warsaw, Poland) for 15 seconds. Subsequently, the seeds were disinfected with a 1.5% sodium hypochlorite solution (Merck Millipore, Warsaw, Poland), shaken for 15 minutes, and rinsed three times with sterile water. The seeds were sown on an autoclaved Murashige and Skoog (MS) medium (Murashige and Skoog, 1962) in either liquid or solidified form with agar (Agar-agar for microbiology, 8–9 g/L) (Merck Millipore, Warsaw, Poland). The culture was conducted in 250–300 mL Erlenmeyer flasks with 100 mL medium. During the passage on fresh media, dead parts of plant material were separated, and clumps were torn apart or transferred completely to a larger 750 mL Erlenmeyer flask containing 100 mL of medium.

### 2.2 *In vitro* culture of *Arnica* taxa

*In vitro* cultures of the studied taxa were conducted on MS medium at full strength, containing 30 g/L of sucrose (POCH, Gliwice, Poland) in either liquid form or solidified with agar. The pH of the medium was set to 5.8 before autoclaving at 121°C for 20 minutes. Shoot tips from previously isolated sterile growing plants were inoculated onto the medium. For the shoot tips culture, a medium containing cytokinin (0.5-1.0 mg/L 6-benzylaminopurine (BAP)) (Merck Millipore, Warsaw, Poland) and auxin (0.1 mg/L indole-3-butyric acid (IBA)) (Merck Millipore, Warsaw, Poland) was used. After multiplying the desired number of shoots, two preparatory cycles were initiated. The plants were transferred to hormone-free media. In all experiments, 4 explants were inoculated per Erlenmeyer flask; the explants were small rosettes containing 2–3 roots, with an initial weight of 0.9 ± 0.1 g. The cultures were maintained in phytotron chambers for 6–8 weeks at a temperature of 20 ± 2°C with a photoperiod of 16/8 h light/dark at photosynthetic photon flux density (PPFD) 30-150 µmol m^-2^ s^-1^, depending on the experiment. PPFD values were verified using OPTEL phytophotometer, FR-10 (Sonopan, Białystok, Poland). All experiments were carried out in a phytotron equipped with a cooling system and lighting from 120 fluorescent lamps, from a mix of Day Light, Cool Light, and Flora lamps (Philips, Poland) placed from above and installed on two frames in a ceiling separated from the main chamber by plexiglass and armed with an independent exhaust system. Qualitative analysis of SLs was performed using high-resolution liquid chromatography with quadrupole time-of-flight mass spectrometry (LC-QTOF-MS) (Waters, Milford, MA, USA) (Parafiniuk et al., 2023).

#### 2.2.1 Culture elicitation

To enhance the biosynthesis of SLs, *Arnica montana* and variety Arbo were elicited with jasmonic acid (JA) (Merck Millipore, Warsaw, Poland) and chitosan (Merck Millipore, Warsaw, Poland) at different concentration ranges. The plant cultures were maintained on MS medium solidified with agar, without plant hormones in a phytotron for 6 weeks without the addition of elicitors at 20 ± 2°C with a photoperiod of 16/8 h light/dark at a PPFD of 70 µmol m^-2^ s^-1^. After 6 weeks of culture, plants were transferred into a liquid MS medium with different concentrations of chitosan aqueous solutions and alcoholic solutions of JA. Sterile, filtered JA solution was prepared in 70% ethanol and diluted to final concentrations of 0.25, 0.50, 1.0, and 1.5 mg/L. Chitosan solution was prepared in sterile water to final concentrations of 50, 100, 150, 200, and 250 mg/L; pH was regulated prior to steam autoclaving and then added to the propagated plant cultures. The control in chitosan experiments was pure liquid MS medium. In the JA study, the medium supplemented with 70% ethanol in the same volume as in the case of JA was used. Cultures with elicitors were maintained in a phytotron for the next 7 days, extracted, and then the qualitative content of SL was analyzed by LC-QTOF-MS.

#### 2.2.2 Light treatments

To investigate the effect of light wavelength on the biosynthesis of SLs, the propagated explants were exposed to four light spectra: yellow (570 nm), blue (465 nm), red (660 nm), and the full spectrum of light. The PPFD was set at the same level of 30 μmol m^-2^ s^-1^. The experiment was carried out in experimental cabinets using Philips fluorescent lamps emitting red light (‘TL’D 36W/15), blue light (‘TL’D 36W/18), yellow light (‘TL’D 36W/16), and full spectrum light (‘TL’D 36W/84). The air-conditioning was used to establish the same temperature in each cabinet with a separate conditioning system at 20 ± 2°C with a photoperiod of 16/8 h light/dark. The cultures were maintained in cabinets for 6 weeks.

Studies on the influence of PPFD on the biosynthesis of SLs were carried out, three-photon flux densities of 50, 100, and 150 μmol m^-2^ s^-1^ were used, and subsequently, qualitative analysis of SLs was carried out.

### 2.3 Bioreactor for the plant of *A. montana* ‘Arbo’

To scale up the process, six fused clumps of 4 weeks cultured plants of *A. montana* ‘Arbo’ with a developed root system and an initial mass of 80 ± 3 g were transferred to 5-liter colorless polypropylene containers containing 250 mL of liquid MS medium lacking plant hormones. In the fourth week of cultivation, 250 ml of fresh sterile medium was added. Cultures were maintained in growth chambers for 9 weeks at 20 ± 2°C with a 16/8 h light/dark photoperiod at PPFD of 100 µmol m^-2^ s^-1^. Bioreactors were prepared in triplicate. The composition of SLs was analyzed using the LC-QTOF-MS method.

### 2.4 Extraction and quantitative analysis of SLs using LC-QTOF-MS

The extraction procedure was performed according to the method described in Parafiniuk et al. (2023). Samples were prepared in triplicate, and concentrations were calculated per unit dry weight of the sample. Qualitative analysis of SLs was performed using LC-QTOF-MS. The LC system consisted of a Waters nanoACQUITY UPLC (ACQUITY UPLC I-Class, Waters, Milford, MA, USA) and a nanoACQUITY HSS T3 column (C18 phase, 1 mm i.d., 50 mm length, 1.8 µm particle size). Eluates were analyzed using a quadrupole time-of-flight mass spectrometer (Xevo G2 QTOF MS, Waters, Milford, MA, USA) and were equipped with an electrospray ionization (ESI) source. Calibration standards were prepared using the previously validated and described method by Parafiniuk et al. (2023). The standards of (-)-α-santonin were obtained from Merck Millipore (Warsaw, Poland), and helenalin was sourced from Cayman (Michigan, USA). Ethanol, methanol, formic acid, and water were obtained from Merck Millipore (Warsaw, Poland), and leucine-enkephalin was acquired from Waters (Milford, MA, USA).

### 2.5 Statistical analysis

Parametric data were analyzed using one-way analysis of variance (ANOVA). Results are expressed as means ± standard deviations, based on three independent replicates. A *p*-value of less than 0.05 (*p* < 0.05) was considered statistically significant. All statistical analyses were performed using Statistica software, version 12.0 (StatSoft Inc., Kraków, Poland).

## 3 Results

### 3.1 *In vitro* culture

*In vitro* cultures of the studied *Arnica* taxa were initiated by sowing seeds, with the germinated seedlings, along with the subsequently growing plants, propagated through division on hormone-free media. The passaged explants should contain a shoot with fragments of the rhizome and roots, as smaller explants, such as single buds, failed to grow in half of the cases. Cultures can also be propagated by passing longer shoots placed horizontally on the medium, which encourages the development of more axillary buds.

**Figure 1:**
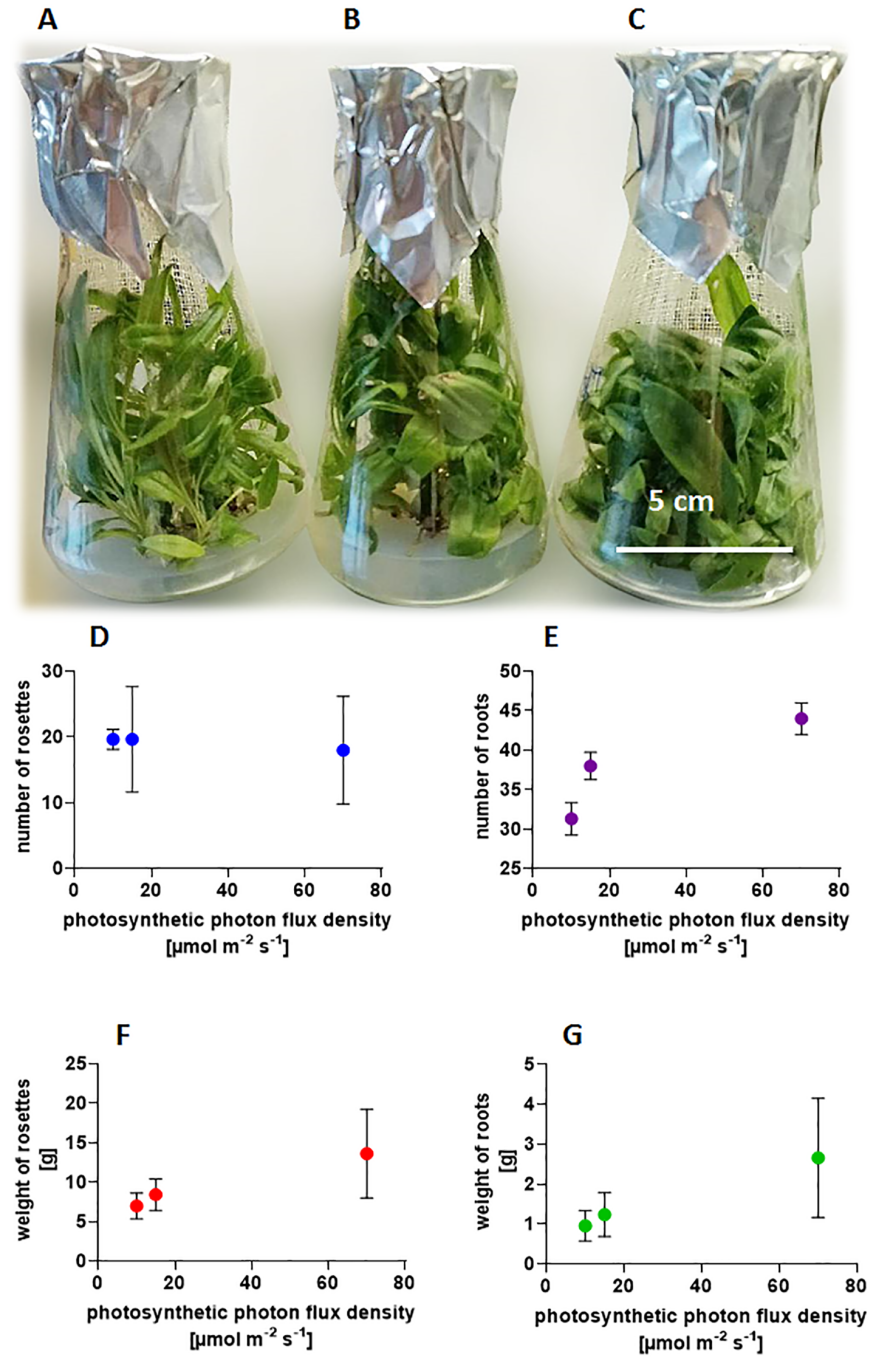
Influence of light intensities **(A)** 10 µmol m^-2^ s^-1^; **(B)** 15 µmol m^-2^ s^-1^; **(C)** 70 µmol m^-2^ s^-1^ on plant morphology –photography of *Arnica montana* ‘Arbo’ cultivated at 20 ± 2°C with a photoperiod of 16/8 h light/dark, after 12 weeks of culture, and on graphs: the number of **(D)** shoot buds; **(E)** roots; **(F)** weight of rosettes; and **(G)** roots. Experiments were performed in 20 replicates, five flasks contained four inoculated small buds with roots, with an initial weight of 0.370-0.5 g each.

To evaluate weight changes throughout the development cycle under different light conditions, the cultures were initiated with small rosettes featuring 2–3 roots and an initial weight of 0.8-1.0 g. A new root system formed at the base of the axillary shoots as they emerged from dormant buds. The shoot weight increased rapidly. After 12 weeks, the older leaves turned yellow due to nutrient deficiencies in the medium, particularly in the flask where plants have developed many shoots. The increases in fresh weight of the cultures are impressive, ranging from 6 to 10 times, and are influenced by light conditions to which the culture is exposed (**Figure 1**). Six rosettes originating from three buds developed at a PPFD of 10 µmol m^-2^ s^-1^(**Figure 1A**), resulting in a total mass of 2.698 g FW. At 15 µmol m^-2^ s^-1^ (**Figure 1B**) the average number of rosettes rose to 9.5 with a combined mass of 5.022 g FW, while in cultures illuminated by 70 µmol m^-2^ s^-1^(**Figure 1C**), the number of rosettes grew to 12 with a total weight of 8.931 g FW. The experiment (**Figure 1**) shows that the length and width of leaves change under the influence of light, but not the total number of rosettes (**Figure 1D**), while the number of roots increases (**Figure 1E**). Light intensity affects the weight of roots (**Figure 1G**) and the size of individual rosettes (**Figure 1F**). Here, we are dealing with the form-forming effect of light and better leaf development, which is reflected in the development of secretory structures on their surface and the content of SLs.

Increasing light exposure significantly alters the shape of the leaves, causing them to become visibly shorter and wider in both studied taxa (**Figure 1**). The poor lighting of the culture results in weaker development of covering hairs visible under a stereoscopic microscope, whereas higher light intensity promotes their formation. Although non-glandular trichomes are not uniformly visible on all leaves, likely due to uneven light distribution within the flask, caused by the overlapping leaves and numerous rosettes.

In the conducted study, we quantitatively and qualitatively analyzed the content of secondary metabolites from the group of SLs, specifically focusing on helenalin and dihydrohelenalin esters. We compared these metabolites in plants grown in the field with those cultivated *in vitro* under laboratory conditions. Preliminary analyses of the SLs in cultured plants showed promising results. In field-growing plants, it has been shown that these compounds accumulate in the leaves (0.85 mg/g DW in the case of *A. montana*; 8.40 mg/g DW in *A. montana* ‘Arbo’). At the same time, the short shoots and roots themselves contain trace amounts of lactones (Parafiniuk et al., 2023). Because the SL content in the leaves of plants grown on MS medium was several times higher than in field-grown plants, we attempted to determine what chemical factors in the medium and the culture’s environmental conditions significantly impact the level of biosynthesis of these compounds. We investigated whether *in vitro* culture of *Arnica* taxa could be a viable alternative to field farming for producing these pharmaceutically important secondary metabolites.

In summary, a comparison of SL concentrations in plants grown in soil versus those grown *in vitro* showed that these compounds are significantly more abundant in culture conditions. The concentration of these components increased almost 28-fold in *A. montana* and more than 7-fold in the variety Arbo (50 µmol m^-2^ s^-1^).

A comparison of the two tested taxa of *Arnica montana* showed a significantly higher lactone content in the Arbo variety, which has a much better propagation rate both *in vitro* and under field conditions. The rhizomes and roots of the species from the natural site cultivated *in vitro* are dark and hard, contain less water, and have better-developed supporting tissues, while the Arbo variety has rhizomes with longer internodes, softer with the cork tissue less developed. These observations suggest hormonal differences, primarily cytokinins and in the proportions of GA and ABA, which act antagonistically to each other and are produced by the same biosynthetic pathway as terpenes. Alternatively, higher levels of phenolic compounds may contribute to this characteristic of the wild species’ features. Observations of *Arnica* taxa and results of chemical analyses allow us to conclude that higher levels of lactones are responsible for the reproductive success of the Arbo variety.

#### 3.1.1 Biosynthesis of SLs in subsequent leaves (80 µmol m^-2^ s^-1^) *in vitro*

**Figure 2:**
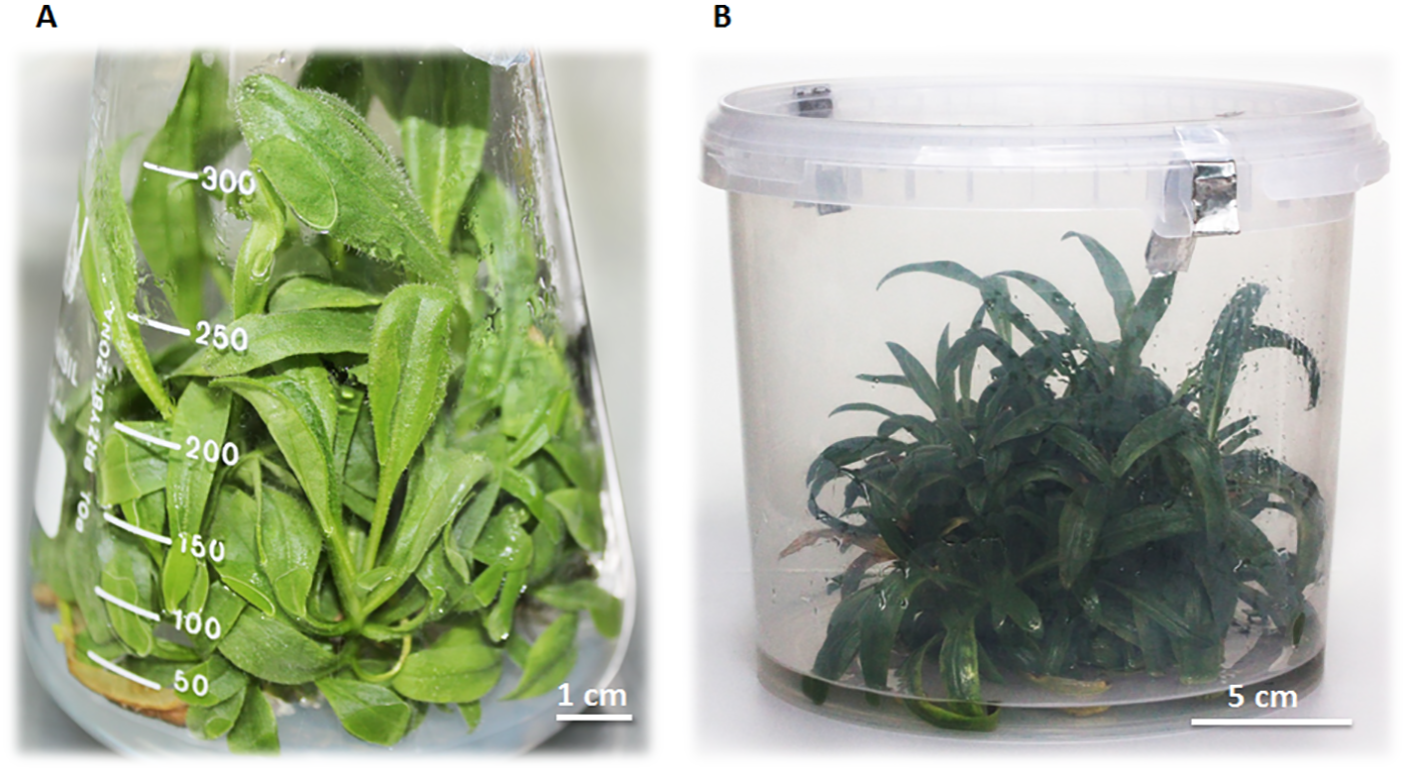
**(A)** *In vitro* culture of *A*. *montana* variety Arbo on agar-solidified MS medium without hormones after 8 weeks of cultivation; **(B)** *A*. *montana* ‘Arbo’ plant bioreactor with liquid MS medium without hormones after 9 weeks of cultivation, under 100 µmol m^-2^ s^-1^, at 20 ± 2°C with a photoperiod of 16/8 h light/dark.

The SL concentrations in leaves of *A. montana* showed a marked increase, with the second and fifth leaves having double the amount of lactones found in the first leaf, and the third and fourth leaves displaying a threefold increase. The analysis shows that the level of lactones along the axis of the shoot is highest in the third and fourth leaves and the lowest in the first leaf. The distribution of lactones is diverse in the *Arnica montana* ‘Arbo’ (**Figure 2A**), where most lactones are found in the first leaf, with a gradual reduction in concentration in the leaves of the next examined nodes. In both taxa, the level of DHM changes the most significantly, with a decrease in variety and with a rise in the wild species leaves until the third node, whereas much smaller fluctuations were recorded in the case of tigloyldihydrohelenalin (DHT); however, overall, all derivatives follow the trends in the total SLs concentration.

#### 3.1.2 Expanding the scale of cultures *in vitro* (100 µmol m^-2^ s^-1^)

Experiments were performed on a liquid medium that accelerates plant growth. After 9 weeks in the bioreactor of *Arnica montana* ‘Arbo’ (**Figure 2B**), the plant reached the fresh mass of 390.2 g, representing nearly a fivefold increase, with a moderate lactone content. The average SL component in the leaves was lower (40.25 mg/g DW) (**Table 1**) than in the flasks culture (64.2 mg/g DW) (**Table 2**), which may be the consequence of less transparency of the container walls, which reduced the light intensity by 20% and cut off wavelengths below 400 nm. It is also worth noting that the resulting clump is not built of leaves themselves; the analysis of the long, older shoots revealed the presence of lactones at 11.93 mg/g DW, whereas the apical shoots and short adventitious shoots contained 14.69 mg/g DW of these components (**Table 3**). In contrast to aerial plant parts, the roots contained only small amounts of lactones (4.07 mg/g DW) (**Table 3**). Additionally, access to light for plants growing in a compact clump is limited. The differences in the amount of lactones in various organs affect the efficiency of the *in vitro* bioreactor. In a 9-week cycle, it was estimated that leaves constitute 84% of the total mass, with shoots and roots 16%, which reduces the total productivity of the bioreactor. The efficiency of the bioreactor from *Arnica montana* ‘Arbo’ plants is 1319.40 mg/g DW of SLs calculated per leaf from one bioreactor’s 9-week cycle. This means that one cycle initiated from 80 g fresh weight of plants yields SLs equivalent to 156.97 g DW of field-grown leaves and 38.26 g DW of flowers. The underground parts of the plant - roots and rhizomes are a rich source of specific essential oils with thymol and its derivatives, which also possess therapeutic and pharmacological properties.

**Table 1:** Distribution of sesquiterpene lactones [mg/g DW ± SD] from leaves from the subsequent nodes from *A. montana* ‘Arbo’ plants cultivated in the bioreactor illuminated with photosynthetic photon flux density - 100 µmol m^-2^ s^-1^.

*A. montana* ‘Arbo’					
SLs	first leaf	second leaf	third leaf	fourth leaf	fifth leaf
DH	1.76 ± 0.17	1.63 ± 0.90	1.37 ± 0.46	1.27 ± 0.13	1.10 ± 0.28
H	0.41 ± 0.32	0.38 ± 0.33	0.25 ± 0.31	0.08 ± 0.10	0.05 ± 0.03
DHA	1.86 ± 1.11	1.52 ± 1.12	1.24 ± 0.83	1.09 ± 0.14	0.89 ± 0.43
HA	0.36 ± 0.23	0.30 ± 0.16	0.27 ± 0.14	0.20 ± 0.03	0.21 ± 0.12
DHM	28.57 ± 4.29	26.18 ± 4.18	24.26 ± 5.44	25.12 ± 0.65	26.29 ± 1.97
HM	1.86 ± 0.75	1.63 ± 0.50	1.58 ± 0.94	1.14 ± 0.06	1.28 ± 0.57
DHIB	0.78 ± 0.29	0.66 ± 0.27	0.70 ± 0.37	0.49 ± 0.17	0.72 ± 0.15
HIB	2.96 ± 0.02	2.64 ± 0.01	2.82 ± 0.73	2.71 ± 0.01	2.99 ± 1.40
DHT	3.47 ± 0.90	3.10 ± 1.02	3.17 ± 1.91	3.11 ± 0.58	2.87 ± 0.92
HT	0.74 ± 0.42	0.56 ± 0.17	0.51 ± 0.25	0.41 ± 0.17	0.61 ± 0.31
DHMB/DHIV	2.03 ± 0.86	1.79 ± 0.52	1.85 ± 0.82	1.69 ± 0.41	1.69 ± 0.61
HIV/HMB	0.55 ± 0.22	0.44 ± 0.08	0.41 ± 0.13	0.30 ± 0.01	0.35 ± 0.22
Total H	6.90 ± 1.92	5.95 ± 1.21	5.84 ± 2.51	4.84 ± 0.38	5.49 ± 0.21
Total DH	38.48 ± 7.63	34.87 ± 8.01	32.59 ± 9.83	32.78 ± 2.07	33.55 ± 4.36
Total SLs	45.37 ± 9.55	40.82 ± 9.23	38.43 ± 12.33	37.62 ± 2.45	39.04 ± 4.15

SLs, sesquiterpene lactones; H, helenalin; DH, dihydrohelenalin; HA, acetylhelenalin; DHA, acetyldihydrohelenalin; HM, methacryloylhelenalin; DHM, methacryloyldihydrohelenalin; HIB, isobutyrylhelenalin; DHIB, isobutyryldihydrohelenalin; HT, tigloylhelenalin; DHT, tigloyldihydrohelenalin; HMB, 2-methylbutyrylhelenalin; DHMB, 2-methylbutyryldihydrohelenalin; HIV, isovalerylhelenalin; DHIV, isovaleryldihydrohelenalin; measurement uncertainty U=18.82; n=3; - LOD, below to the limit of detection; SD, standard deviation; ANOVA one-way; significant differences at p < 0.05 (p=0.0243).

**Table 2:** Influence of light intensity photosynthetic photon flux density in the range 50 -150 µmol m^-2^ s^-1^ on lactones content and distribution of helenalin and 11α,13-dihydrohelenalin derivatives [mg/g DW ± SD] in cultured *in vitro* plants of *Arnica montana* and variety Arbo after 8 weeks of culture.

Light treatment (PPFD)	*Arnica montana* ‘Arbo’			*Arnica montana*		
SLs	50 µmol m^-2^ s^-1^	100 µmol m^-2^ s^-1^	150 µmol m^-2^ s^-1^	50 µmol m^-2^ s^-1^	100 µmol m^-2^ s^-1^	150 µmol m^-2^ s^-1^
DH	0.69 ± 0.04	0.80 ± 0.13	1.33 ± 0.08	0.69 ± 0.19	1.03 ± 0.23	1.17 ± 0.28
H	0.43 ± 0.00	0.39 ± 0.05	0.47 ± 0.05	0.07 ± 0.01	0.07 ± 0.00	0.10 ± 0.02
DHA	3.34 ± 0.09	3.39 ± 0.64	5.36 ± 0.29	0.33 ± 0.08	0.42 ± 0.15	0.49 ± 0.15
HA	1.04 ± 0.04	0.80 ± 0.10	0.69 ± 0.12	0.00 ± 0.00	0.00 ± 0.01	0.00 ± 0.00
DHM	24.47 ± 1.06	26.93 ± 9.50	31.56 ± 4.68	13.66 ± 3.94	16.33 ± 4.63	17.68 ± 6.48
HM	4.83 ± 0.25	4.02 ± 0.85	2.50 ± 0.50	0.26 ± 0.04	0.27 ± 0.16	0.30 ± 0.03
DHIB	6.47 ± 0.86	8.32 ± 3.56	7.40 ± 1.20	1.16 ± 0.72	1.54 ± 1.37	1.66 ± 1.06
HIB	1.10 ± 0.10	0.62 ± 0.13	0.26 ± 0.00	0.08 ± 0.08	0.13 ± 0.06	0.00 ± 0.00
DHT	6.08 ± 0.77	6.77 ± 0.71	11.55 ± 1.52	3.81 ± 0.07	5.94 ± 1.04	6.21 ± 1.13
HT	2.21 ± 0.01	1.72 ± 0.69	1.59 ± 0.20	0.20 ± 0.03	0.22 ± 0.05	0.19 ± 0.04
DHMB/DHIV	6.85 ± 0.40	8.85 ± 0.53	10.17 ± 2.21	3.54 ± 0.03	4.62 ± 1.57	4.70 ± 0.05
HIV/HMB	1.99 ± 0.01	1.57 ± 0.21	1.02 ± 0.23	0.06 ± 0.02	0.05 ± 0.04	0.08 ± 0.01
Total H	11.60 ± 0.19	9.13 ± 1.77	6.53 ± 1.09	0.66 ± 0.17	0.74 ± 0.31	0.67 ± 0.02
Total DH	47.90 ± 1.50	55.08 ± 15.07	67.37 ± 9.24	23.18 ± 5.03	29.89 ± 6.92	31.90 ± 9.15
Total SLs	59.50 ± 1.31	64.20 ± 16.84	73.90 ± 10.33	23.84 ± 4.83	30.63 ± 7.23	32.57 ± 9.17

H, helenalin; DH, dihydrohelenalin; HA, acetylhelenalin; DHA, acetyldihydrohelenalin; HM, methacryloylhelenalin; DHM, methacryloyldihydrohelenalin; HIB, isobutyrylhelenalin; DHIB, isobutyryldihydrohelenalin; HT, tigloylhelenalin; DHT, tigloyldihydrohelenalin; HMB, 2-methylbutyrylhelenalin; DHMB, 2-methylbutyryldihydrohelenalin; HIV, isovalerylhelenalin; DHIV, isovaleryldihydrohelenalin; measurement uncertainty U=18.82; n=3; - LOD, below to the limit of detection; ANOVA one-way; significant differences at p < 0.05 (p=0.0216).

**Table 3:** Sesquiterpene lactones content [mg/g DW ± SD] in plant organs from short and long adventitious shoots, and roots of *A. montana* ‘Arbo’ plants cultivated in the bioreactor illuminated with photosynthetic photon flux density - 100 µmol m^-2^ s^-1^.

*A. montana* ‘Arbo’			
SLs	long adventitious shoots	short adventitious shoots	roots
DH	0.05± 0.06	0.23±	0.06± 0.03
H	0.00± 0.01	0.05±	0.00± 0.00
DHA	0.01± 0.01	0.53±	0.00± 0.01
HA	0.00± 0.00	0.12±	0.00± 0.00
DHM	9.68± 3.45	9.18±	3.08± 0.02
HM	0.15± 0.05	0.56±	0.11± 0.06
DHIB	0.02± 0.03	0.00±	0.00± 0.00
HIB	1.93± 0.25	1.82±	0.69± 0.06
DHT	0.10± 0.01	1.12±	0.06± 0.01
HT	0.00± 0.00	0.20±	0.01± 0.00
DHMB/DHIV	0.00± 0.00	0.67±	0.05± 0.01
HIV/HMB	0.01± 0.01	0.19±	0.02± 0.00
Total H	2.08± 0.33	2.95±	0.82± 0.00
Total DH	9.85± 3.55	11.74±	3.25± 0.04
Total SLs	11.93± 3.22	14.69±	4.07± 0.04

SLs, sesquiterpene lactones; H, helenalin; DH, dihydrohelenalin; HA, acetylhelenalin; DHA, acetyldihydrohelenalin; HM, methacryloylhelenalin; DHM, methacryloyldihydrohelenalin; HIB, isobutyrylhelenalin; DHIB, isobutyryldihydrohelenalin; HT, tigloylhelenalin; DHT, tigloyldihydrohelenalin; HMB, 2-methylbutyrylhelenalin; DHMB, 2-methylbutyryldihydrohelenalin; HIV, isovalerylhelenalin; DHIV, isovaleryldihydrohelenalin; measurement uncertainty U=18.82; n=3; - LOD, below to the limit of detection; SD, standard deviation; ANOVA one-way; significant differences at p < 0.05 (p=0.034).

In summary, the bioreactor productivity of the applied type is 4025.02 mg SLs per 100 g of dry weight of leaves.

### 3.2 Influence of physical factors

#### 3.2.1 Light spectrum (30 µmol m^-2^ s^-1^)

**Figure 3:**
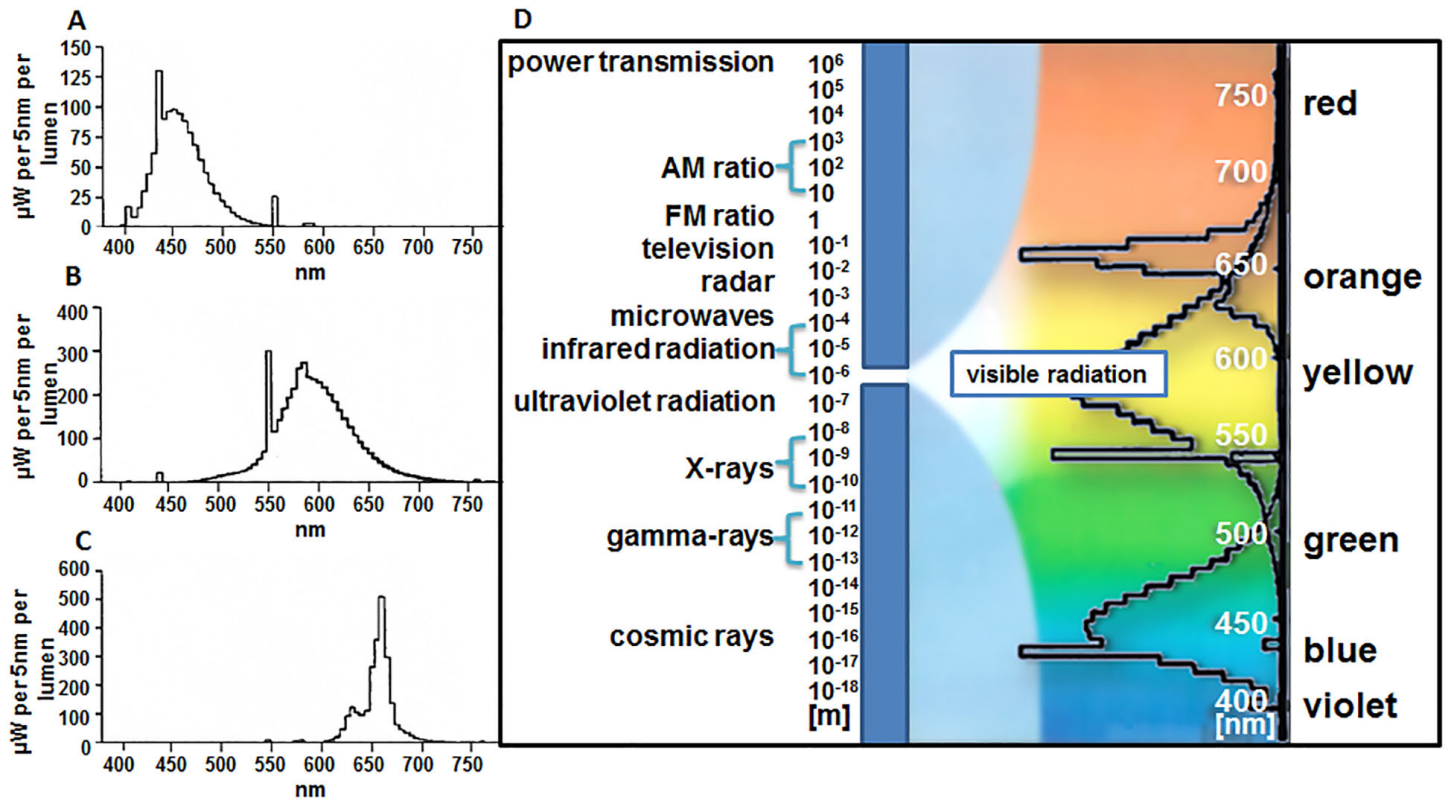
Spectral characteristics of Philips fluorescent lamp emitting various light wavelengths: **(A)** blue, ‘TL’D 36W/18 (465 nm); **(B)** yellow, ‘TL’D 36W/15 (570 nm); **(C)** red, ‘TL’D 36W/15 (660 nm); **(D)** combined tested spectra. Data based on information presented by the producer (Philips, Poland).

The experiments examined the influence of the light spectrum of different wavelengths on the production of SLs and the morphology of plants cultivated under these conditions. White, yellow, red, and blue light were used with the same PPFD intensity, i.e., expressed in micromoles per m^-2^ s^-1^. Preparing appropriate chambers for culture growth required cooling systems and ventilators of different power to obtain the same temperature of 20 ± 2°C and at PPFD of 30 µmol m^-2^ s^-1^. The spectral characteristics of the Philips fluorescent lamps, emitting different wavelengths of light, are shown in **Figure 3**.

**Figure 4:**
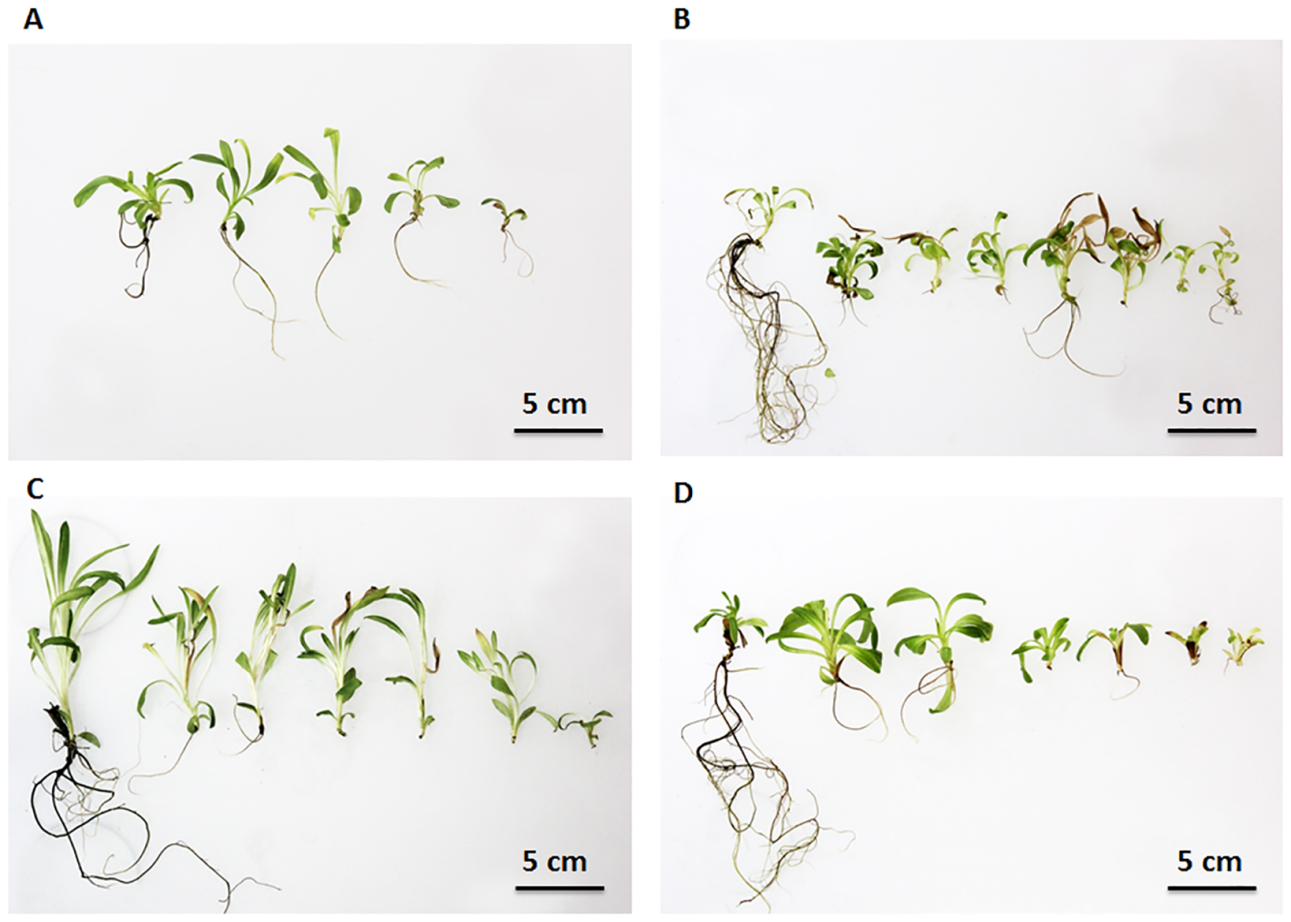
Morphology of *A*. *montana* ‘Arbo’ plants growing under the light of different wavelengths: **(A)** white (full range of the light spectrum); **(B)** yellow (with a maximum at 570 nm); **(C)** red (with a maximum at 660 nm); **(D)** blue (with a maximum at 465 nm), under 30 µmol m^-2^ s^-1^, at 20 ± 2°C with a photoperiod of 16/8 h light/dark, after 6 weeks of culture.

Among the wavelengths used, only red light induced the biosynthesis of SLs, both in the wild species and the variety. In this light spectrum, *A. montana* synthesizes three times more lactones after 2 months of culture, while an increase of over 60% is detected in ‘Arbo’ compared to controls. In wild species, dihydrohelenalin esters predominate, while helenalin derivatives are absent. In a variety, significantly more helenalin esters are synthesized in red light, mainly methacryloylhelenalin (HM) and tigloylhelenalin (HT). The lowest lactone synthesis occurs in the blue part of the spectrum. The morphogenetic influence of the light spectrum (**Figure 4**) was manifested in the diverse development of leaves, the length of petioles, the intensity of rhizome growth, and the number and color of roots. Visually, plants cultivated under white (**Figure 4A**) and blue light (**Figure 4D**) contain the most chlorophyll, but they vary in the shape of the leaves, which are more elongated under white light than those exposed to blue light, and the formation of roots is also prolonged. Plants cultivated under red (**Figure 4C**) and yellow light (**Figure 4B**) contained less chlorophyll, but the leaves developed in both conditions had a completely different shape – in yellow light (**Figure 4B**), the blades were small and short, bent away from the stem. In the red part of the spectrum (**Figure 4C**), etiolated blades were juvenile, elongated, and erect. Intensive root development occurred on shoots growing under white light (**Figure 4A**), and the weakest under red light (**Figure 4C**). Yellow light (**Figure 4B**) promoted shoot branching the most, and white light (**Figure 4A**) the least of all applied conditions.

#### 3.2.2 PPFD intensity (50-150 µmol m^-2^ s^-1^)

By analyzing the *in vitro* profiles of SLs in the studied *Arnica* taxa, it was proven that our native species *A. montana*, is characterized primarily by dihydrohelenalin and its derivatives (97.23%) (**Table 2**). In comparison, in *A. montana* ‘Arbo’, these derivatives are slightly lower (80.5%), and, instead, with a larger fraction of helenalin and its esters (**Table 2**).

Extraction and analyses performed using the LC-QTOF-MS on plant leaves cultivated on MS medium at PPFD of 50, 100, and 150 µmol m^-2^ s^-1^ show significant differences in their ability to biosynthesize SLs compared to the lowest used light intensity. In *A. montana*, it is 23.84 mg/g DW; in the Arbo variety, it is more than twice as high (59.5 mg/g DW) (**Table 2**). It has been shown that the highest light level intensifies the biosynthesis of SLs by 24% in the wild species *A. montana* occurring in Lower Silesia and by 36.6% in the Arbo variety. Growing photon flux density in the *Arnica montana* ‘Arbo’ reduces the synthesis of helenalin derivatives, while it does not affect their synthesis in the *A. montana* wild species. In both taxa, unbound with carboxylic acid, dihydrohelenalin gradually rises with the increasing photon flux density, likely serving as a precursor to all its ester derivatives. As the light energy increases in *A. montana* ‘Arbo’, the content of helenalin esters declines while the analogous dihydrohelenalin esters rise. These findings indicate that light energy contributes to the conversion of helenalin esters into mostly the same dihydrohelenalin esters.

Due to the low concentration of helenalin and its derivatives in *A. montana*, only an increment in the synthesis of isobutyrylhelenalin (HIB), methacryloyldihydrohelenalin (DHM), isovaleryldihydrohelenalin (DHIV), and 2-methylbutyrylhelenalin (HMB) is visible under the growing light intensity, however, the same derivatives occur under these conditions in Arbo variety. Light intensity positively affects the similar profile distribution of SL derivatives, leading to an increased formation of dihydrohelenalin in both examined taxa. As the light fluence rate increases, the distribution profile of these SL derivatives becomes more uniform, further accelerating dihydrohelenalin production in both taxa. Notably, the Arbo variety contains more helenalin than compared to the wild species.

### 3.3 The influence of selected chemical factors

#### 3.3.1 Jasmonic acid (70 µmol m^-2^ s^-1^)

In biotechnology, JA is a known elicitor of the biosynthesis of secondary metabolites, consequently, the effect of this compound added to the agar medium in 4 doses (from 0.25 to 1.5 mg/L) on the SLs in leaves was determined. Preliminary experiments established that the greatest enhancement in the biosynthesis of these metabolites occurs after a short time, 7-day exposure of plants to this hormone. The results obtained after the application of JA indicate that the concentrations of 0.25 – 1.5 mg/L for *A. montana* ‘Arbo’ fit the Gauss curve with an optimum dose of 1 mg/L of nutrient solution, where the level of SLs in leaf tissues enlarges almost twofold compared to the control. In the case of the wild species, the best dose appears to be 0.25 mg/L, which brought about 34.5% more lactones than the leaves of control plants; the remaining concentrations were inferior.

In *A. montana* ‘Arbo’, compared to dihydrohelenalin an increase in the helenalin derivatives was also observed at all concentrations used. At the 0.25 mg/L dose, helenalin esters magnified fourfold, while the total dihydrohelenalin esters increased by 40%. JA induced the greatest formation of methacrylic and isovaleric derivatives of both helenalin and dihydrohelenalin. In the case of the wild species, such an effect of JA was not demonstrated, and the increased level of lactone correlates with the uniform synthesis of all derivatives of both groups of compounds.

JA, after a seven-day application in culture on MS agar medium, stimulates the biosynthesis of SLs, wild species respond already at a dose of 0.25 mg/L, and the variety requires a higher concentration of 1 mg/L. In *A. montana* ‘Arbo’, this growth regulator enhances biosynthesis by 1.5 times and changes the proportions between helenalin and dihydrohelenalin esters, promoting helenanolides biosynthesis. It can be assumed that the wild species originating from a site in southwestern Poland in the Izera Mountains is more sensitive to the JA used, and the optimal effect occurs at lower doses of this hormone.

#### 3.3.2 Chitosan (70 µmol m^-2^ s^-1^)

Another well-known elicitor used in *in vitro* cultures is chitosan, a polysaccharide resulting from the deacetylation of chitin (poly-β-1,4-D-glucosamine). This compound was used in 5 doses (50–250 mg/L agar MS medium) for the *Arnica* species and variety. The tested plants responded with increased biosynthesis of SLs to the application of chitosan in the medium, and these effects were visible after 7 days of culture. The sensitivity of each taxon to the doses of chitosan varies. For the wild form of *Arnica montana*, the optimal concentration for lactone production is 50 mg/L, while for the Arbo variety, it is 100 mg/L. In the case of the wild species, the lactone content was elevated fivefold compared to the control on the medium without chitosan. From among all lactones, this polymer mainly stimulated the biosynthesis of dihydrohelenalin derivatives, i.e., DHM, isobutyryldihydrohelenalin (DHIB), DHT, and 2-methylbutyryldihydrohelenalin (DHMB), isovaleryldihydrohelenalin (DHIV). For the *A. montana* ‘Arbo’, the most effective concentration was 100 mg/L, where the contents of formed lactones amplified almost twice, and the ratio of helenalin esters to dihydrohelenalin changed from 1:5 to 1:2.5, and helenalin and dihydrohelenalin derivatives are synthesized evenly. At all doses of chitosan, except the highest, the total lactones level is higher than in the control, whereas in the wild species, it is equal to or lower than in the control. It is worth adding that both elicitors (JA and chitosan) induce rapid reactions; however, the remarkable effect diminishes with prolonged exposure. JA serves as an effective induction factor for the Arbo variety, while applying chitosan significantly increases the synthesis of SLs in *A. montana*, resulting in a fivefold higher concentration. The observed data suggest the effectiveness of chitosan, a factor imitating the attack of fungal pathogens and insects, and primarily highlights the different sensitivity of both taxa to the elicitors used.

## 4 Discussion

### 4.1 *In vitro* cultures of *Arnica* taxa

*In vitro* technology enables the production of desirable secondary metabolites with pharmaceutical applications and high commercial value. In the case of *A. montana*, attempts have been made to produce bioactive secondary metabolites in calli, shoots, transgenic roots, and plants (Malarz et al., 1993; Schmidt et al., 1998; Weremczuk-Jeżyna I and Wysokińska, 2006; Petrova et al., 2014; Stefanache et al., 2014). In most *in vitro* studies, either alone or in combination with auxin, cytokinins are applied to achieve high reproduction rates. The addition of cytokinin enhances the reproduction rate while decreasing the size of the leaf blade, resulting in an increased number of shoots with smaller leaves. Since previous studies (Malarz et al., 1993; Schmidt et al., 1998; Parafiniuk et al., 2023) have demonstrated that leaves are the site of SLs biosynthesis, our study aimed to create conditions promoting leaf blade expansion. This was achieved by culturing shoots on the MS basal medium. At the same time, it turned out that on MS basal medium, plants of both taxa have high reproductive rates of 5 to 7 axillary shoots within 10 weeks per initial explant, with a lower reproductive coefficient for the species *A. montana*. Both taxa showed a substantial five to tenfold expansion in plant fresh weight during the 10-week culture growth cycle.

Our chemical analysis of leaves revealed the presence of helenalin, dihydrohelenalin, and their six esters with carboxylic acid. Contrary to this, *A. montana* plants originating from a natural site in the Izera Mountains and grown in the ground in Wroclaw Botanic Garden did not contain helenalin derivatives, whereas, in the Arbo variety, these derivatives constituted approximately 15% of the synthesized lactones, demonstrating the genetic difference of both taxa as shown by Schmidt (2023). The dihydrohelenalin type predominating in *A. montana* subsp. *montana* is found in Central and Eastern Europe, while the helenalin-rich type is recognized as subsp. *atlantica* on the Iberian Peninsula (Schmidt, 2023).

The main ester synthesized in the leaves of ground-growing plants in both taxa is DHM. However, unlike the species, the Arbo variety contains additional helenalin esters comprising 15% of the total content, with the highest amounts of HM and isovalerylhelenalin (HIV) and HMB (Parafiniuk et al., 2023). In plantlets cultured *in vitro* on MS medium, the identical tendency in the profile of SL synthesis is observed with the same dominant derivatives as in the field. This allows the examined taxa to be classified as separate subspecies.

### 4.2 Comparison of SL levels in *in vitro* culture and field cultivation

For the first time, the relationship between *in vitro* culture and the synthesis of the most important bioactive metabolites in *A. montana* plants was presented by Malarz et al. (1993) The authors demonstrated the production of helenalin acetate (HA) in plants cultured *in vitro*, with this compound being detected primarily in leaves, less so in shoots, and least in roots. Our results fully confirm the relationship between the organ and the level of SL synthesis reported by Malarz et al. (1993), indicating that leaves are the main site of biosynthesis. Schmidt et al. found that plantlets of *Arnica montana* growing in suspension culture yield the same qualitative SL composition but in amounts about 10 times lower than in the field (Schmidt et al., 1998). However, the authors did not confirm the presence of SLs in callus and hairy root cultures. In *A. montana* from the northern region of the Romanian Eastern Carpathians, the total secondary metabolite (SLs) for *in situ* samples ranged from 0.08% to 0.33%. Samples harvested from *in vitro* cultures before the acclimatization stage showed an estimated SL content of 1.15% to 1.29%. After acclimatization *ex vitro*, it rose to 1.15-1.38%, with qualitative traits similar to the flower heads samples (Stefanache et al., 2014).

Moreover, in our study, the biosynthesis of SL in shoots cultured *in vitro* in comparison to field conditions was 30 times higher in *A. montana* and 10 times higher for *A. montana* ‘Arbo’ under increased light intensities (PPFD) in the range of 100-150 µmol m^-2^ s^-1^ (**Table 2**).

The total content of SLs in *A. montana* increased threefold from the 1^st^ to the 3^rd^ leaf and gradually decreased in the subsequent leaves, whereas, in the Arbo variety, the highest level of these substances was noted in the first leaf. This indicates that helenalin and dihydrohelenalin derivatives are linked to the juvenile growth stage, highlighting the significance of these compounds in protecting the young leaves of plants. Helenalin probably undergoes hydrogenation as the leaves develop. Schmidt et al. (1996) showed that helenalin and its esters undergo rapid conformational exchange in solutions, which is responsible for the existing entropy differences depending on the quantitative composition of the equilibrium in solvents with different polarities. The hydrogenation of the α-11,13 double bond leads to the formation of 11,13-dihydrohelenalin, stabilizing the conformer (TC7 or TC10) (Schmidt et al., 1996). This suggests a change in the cell’s environment where lactones are produced, or a shift in the cellular compartments directing these compounds, which enhances the formation of dihydrohelenalin derivatives. More extensive *in vitro* studies of *A. montana* shoots were conducted by Petrova et al. (2014) who applied various types of MS medium and plant support, including agar, liquid, liquid with paper bridges (static paper bridge support), and a TIS (temporary immersion system) RITA^®^ bioreactor on modified forms of MS medium, supplemented with two plant hormones, cytokinin (1 mg/L BA) and auxin (0.1 mg/L indole-3-acetic acid (IAA)). The cultures were maintained in a growth chamber at 25 ± 2°C, with a 16-hour photoperiod of cool-white light (40 µmol m^-2^ s^-1^). The total SL content in the case of the study by Petrova et al. (2014) across various types of culture was 7.62 mg/g DW for the agar medium, 13.81 mg/g DW for the liquid medium with paper bridges, and 15.34 mg/g DW for the RITA^®^ bioreactor. With the results we obtained, the quantity, presented by Petrova et al. (2014) for *A. montana* subsp. *montana* is similar, but for the light intensity of 30 µmol m^-2^ s^-1^, whereas these values are twice as high for light intensity increasing to 150 µmol m^-2^ s^-1^. This points to the rate of appropriate culture illumination in the higher biosynthesis of SLs. Similar to the results of Petrova et al. (2014), our study also confirmed that dihydrohelenanolides predominated in cultured plants, but with significantly lower levels of acetyldihydrohelenalin (DHA) and high contents of DHM.

In our case, the culture is maintained on full-strength Murashige and Skoog (1962) nutrient medium without the addition of hormones, unlike in the studies by Malarz et al. (1993) and Petrova et al. (2014). Such a medium promotes the formation of complete plants, meaning that leaves develop along with rhizomes and roots. The increased production of sesquiterpene lactones is associated with culture conditions that favor the growth and differentiation of whole plants, rather than just the proliferation of shoots without rhizomes and roots. Cytokinins are juvenile hormones that promote proliferation but inhibit maturation and cell differentiation processes; therefore, their use is undesirable when the goal is to achieve high synthesis of secondary metabolites.

The results observed during the experiments indicate that leaves from field cultivation may also be a source of a certain amount of active metabolites. An increase in SLs in ground farming can be achieved by reducing exposure to sunlight by shading with a slightly red polypropylene foil, which should maintain the youngest leaves in the juvenile growth phase. Roofing the crop, but not tunneling, in which the temperature will increase significantly (mountain plant), will additionally protect the glands on the leaf surface from lactones washing out by rain, because these compounds are slightly soluble in water (0.2 mg/ml, Cayman Chemical Company, (2022)). Field yields will not reach the values obtained *in vitro*, where sucrose is applied as a carbon source, but the lactone content in leaves should increase. Reducing lighting will reduce photosynthetic efficiency, weakening growth. Therefore, it will be important to maintain the equilibrium between lactone biosynthesis and the proper growth intensity. In the case of flower plantations, these recommendations do not apply because photosynthetic efficiency and the accumulation of primary metabolites in the rhizome in the year preceding flowering determine the number of inflorescences and flower heads on the shoot.

### 4.3 Influence of physical factors

#### 4.3.1 Light intensity, PPFD (50 - 150 µmol m^-2^ s^-1^)

Light affects the quality and efficiency of *in vitro* culture through spectrum, irradiance, and exposure length. It has been shown that an increase in photon flux density correlates with higher SL content in leaf tissues (**Table 2**) The radiation sources used in our study *in vitro* were fluorescent lamps emitting white light or broadband spectra of blue, yellow, and red. Experiments conducted at the light intensities (PPFD) of 50, 100, and 150 µmol m^-2^ s^-1^ with a 16-hour photoperiod demonstrated that higher photon flux densities correlate with increased SL content in leaf tissues (**Table 2**). Usually, light intensities typically used in *in vitro* culture range from 20 to 30 µmol m^2^ s^-1^, resulting in low SL content. Light has a formative effect on the development of *Arnica* plants by promoting the enlargement of the leaf blade and enhancing the formation of secretory structures on the leaf surface where lactones should be synthesized.

The positive impact of light on the biosynthesis of lactones in sunflower seedlings was demonstrated by Spring et al. (1986), who found that as light intensity increased to 100 W m^2^ (approximately 267.36 µmol m^-2^ s^-1^), the concentration of SLs (niveusin C and 15-hydroxy-3-dehydrodesoxyfruticin) rose six-fold. However, further increments in fluency rates over 100 W m^2^ did not lead to additional accumulation of lactones. The light-induced production of 15-hydroxy-3-dehydrodesoxyfruticin occurred after a short lag phase of 40 to 60 min, while the level of niveusin C remained unchanged for the next 12 h of exposure (Spring et al., 1986). Similar findings are reported by (Scavo and Mauromicale, 2020) regarding the phytotoxic lactone cynaropicrin, which synthesis grows under 60% shading in field conditions. Additionally, the SL content in lettuce rises after the plants are covered with foils that block ultraviolet (UV) radiation (Laurenčíková et al., 2023). Previous reports on *Arnica montana* ‘Arbo’ by Spitaler et al. (2006) show that the total contents of SLs and flavonoids were not positively correlated with the altitude of the growth site. Phenolics protect plants from UV radiation, and increased altitude enhances the synthesis of caffeic acid derivatives and specific ortho-hydroxylated flavonoids (Spitaler et al., 2006). These findings indicate that intense sunlight exposure may reduce the synthesis of lactones while enhancing the production of compounds that protect plants from strong solar radiation. Similar observations were made by Stefanache et al. (2014) in their study of Romanian *A. montana*, comparing the phenolic content in wild populations to that cultivated *in vitro*. Their findings revealed that samples from *in vitro* cultures had a low phenolic acid content and high SL levels, whereas wild-growing populations exhibited the opposite trends (Stefanache et al., 2014). Ecologically, SLs manifest antibacterial and antifungal properties and inhibit the biosynthesis of nucleic acids in invading pathogens, which is crucial for protecting susceptible young tissues under low light exposure (Drogosz and Janecka, 2018). Additionally, lactones can interact with plant hormones to promote elongational growth toward the light, as some lactones have been found in shoots at concentrations of 11–14 mg/g DW and in roots at 4 mg/g DW. High light intensities affect plant structure, morphology, and physiological changes associated with tissue maturity. Under full irradiance, specific synthesized metabolites can partially perform the functions of SLs.

It might be asked why the lactone content in field conditions is lower than in the *in vitro* culture, despite the fact that the dry mass of leaves growing in the field is half to three quarters higher, the lactone content is several times lower. We suspect several reasons for this phenomenon: strong insolation promoting rapid maturation and accumulation of phenolic compounds, including thymol, whose biosynthesis occurs from the same precursors (acetate/mevalonic pathway; unpublished data); the action of atmospheric factors: wind, which can damage the structure, and rain, which can aid leakage partially water-soluble lactones from the trichomes on the leaf surface. In summary, our findings and those from various authors indicate a duality in the plant protection system of *A. montana* under different light conditions. Specifically, low light intensity promotes the active synthesis of lactones, while high light intensity, particularly with short wavelengths, encourages the production of phenols and ortho-hydroxylated flavonoids. Additionally, SLs, as a group of substances related to strigolactones, can influence plant growth under varying light conditions, as demonstrated by (Spring et al., 1986).

#### 4.3.2 Influence of the light spectrum on the synthesis of SLs

Both the quantity and quality of light influence photosynthesis and plant morphogenesis, reflecting the multifaceted impact of this factor on the receptor system, including phytochrome, cryptochrome, phototropins, zeaxanthins, and others. Analysis of different light spectra shows a modification in plant morphology (**Figure 4)**. Plants exposed to blue light were shorter than the control group but exhibited a similarly intense green color. In contrast, plants grown under yellow light were more branched, had smaller leaf blades, and showed greater etiolation. Those grown under the red part of the spectrum were the most etiolated, displaying elongated, stretched shoots and narrow, long leaf blades.

In both the wild species and the cultivar, only the red part of the spectrum initiated the rise in SL synthesis by over 63%, leading to an increase in helenalin derivatives in the *Arnica montana* ‘Arbo’ and a threefold increase in dihydrohelenalin esters in *A. montana* species compared to control under the white light of the same photon flux density. A similar effect of red light on sunflowers was evidenced by Spring et al. (1986), furthermore, the action of red light was partially altered by a subsequent far-red pulse. The total synthesis of SLs was also associated with red light in holy basil (Chutimanukul et al., 2022). These findings suggest that SL synthesis is controlled by phytochrome, with the red light-induced form, Pfr, dominating over Pr. This dominance triggers the biosynthesis of SLs, especially in low light conditions (PPFD 30-40 µmol m^-2^ s^-1^).

Phytochromes are built from two polypeptide regions: the photo-sensing domain on the amino-terminal end and the carboxy-terminal module involved in the dimerization of phytochrome polypeptide of histidine kinase-related domain chains. The carboxy-terminal ends in directed nuclear localization and exhibits serine/threonine kinase activity, facilitating the degradation of the transcription factor–phytochrome interaction factor 3 (PIF3), which activates photosynthetic genes in the dark (Sharrock, 2008). The interaction between the C-terminal module and the degradation of the transcription factor causes photobody biogenesis, which generates the regulated phytochrome-mediated signaling and physiological outputs (Matsushita et al., 2003). Consistent with these findings and the model of red light causing photomorphogenesis, Ahn et al. (2022) proposed that phytochrome B (phyB) Pfr is required for the endoplasmic reticulum (ER) stress response *via* induction of expression of ER luminal protein chaperones and unfolded protein response (UPR) genes in the nucleus signaling. In stress reaction, reactive oxygen species (ROS) are accumulated in *Arabidopsis* cells (Ahn et al., 2022). It may be hypothesized that they are responsible for incorporating an oxygen atom in a lactone ring formation, like during the synthesis of leucodin in chicory by cytochrome P450. Furthermore, most cytochrome P450 is also localized in the ER, leading to blue light-reversible inhibition of enzyme activity (de Kraker et al., 2002). Our results support the generally presented statement that red light enhances the accumulation of terpenoids, whereas blue light inhibits terpenoid biosynthesis (Kuo et al., 2015).

Phytochromes, particularly phytochrome A (phyA) and phytochrome B (phyB), are pivotal in mediating plant responses to light, influencing various developmental processes, including seed germination, stem elongation, and flowering. These responses are intricately linked to gibberellin (GA) biosynthesis and signaling pathways (Blázquez and Weigel, 1999). In *Arabidopsis*, red light exposure induces the expression of GA biosynthetic genes, including GA4 and GA4H, which encode GA 3β-hydroxylases responsible for the final steps in GA biosynthesis. Notably, GA4H expression is regulated explicitly by (phyB), whereas GA4 expression is influenced by other phytochromes, indicating a complex regulation of GA biosynthesis by light quality (Yamaguchi et al., 1998). Phytochromes, particularly phyB, can modulate plant responses to GAs by influencing GA sensitivity. For instance, in *Arabidopsis*, phyB mutants exhibit enhanced hypocotyl elongation in response to exogenous GAs, despite having wild-type levels of endogenous GAs. This indicates that phyB may regulate GA-dependent responses by altering GA sensitivity rather than biosynthesis. Phytochromes interact with other phytohormones, such as brassinosteroids (BRs), to regulate GA biosynthesis and signaling. BRs can enhance the transcriptional activity of phytochrome-interacting factors (PIFs), which modulate the expression of GA biosynthetic genes like GA3ox and GA20ox. This crosstalk ensures coordinated regulation of growth processes like cell elongation (Castro-Camba et al., 2022). The interplay between phytochrome signaling and GA biosynthesis is crucial for plants to adapt to changing light conditions. By modulating the expression of key enzymes like CPS and KS, phytochromes integrate light signals with hormonal pathways to regulate growth and development processes. Understanding these mechanisms provides insights into how plants coordinate internal and external signals to optimize their developmental strategies.

Red light converts phytochromes from their inactive Pr to active Pfr form. Pfr accumulates in the nucleus and modulates gene expression by interacting with PIFs, PHYTOCHROME INTERACTING FACTORS, leading to their degradation and releasing the suppression of downstream transcription factors involved in secondary metabolism (Legris et al., 2019). Pfr activation upregulates MYB (PAP1), which promotes anthocyanin biosynthesis by activating CHS, DFR, and ANS genes (Zhang et al., 2023). WRKY33 controls the biosynthesis of camalexin and other phytoalexins (Chen et al., 2022). The bHLH TFs work in concert with MYBs to regulate flavonoid pathway genes.

### 4.4 Influence of chemical factors

#### 4.4.1 Jasmonic acid and chitosan

Both JA and chitosan induced a rapid response in *Arnica* plants, resulting in increased synthesis of SLs. The reaction in *A. montana* was stronger after chitosan treatment than JA. In media containing chitosan, the level of lactones expanded fourfold in the wild species and doubled in the Arbo variety. The wild species *A. montana* was more sensitive to the doses used; the lowest dose of chitosan was 50 mg/L, which caused the maximum response, while the optimum dose for *Arnica montana* ‘Arbo’ was 100 mg/L. Chitosan, a fungal cell wall polysaccharide, is recognized by plants as a microbial factor, triggering defense reactions and stimulating systemic immunity. Iula et al. (2022) studied broad reprogramming induced by molecular elicitors using metabolome analysis of tomatoes as a model plant. The response of tomato plants to chitosan was specific; secondary metabolites of the glucosinolate type, strong defense compounds characteristic to the Brassicaceae family, accumulated in the leaves, while isoprenoid derivatives decreased. These results demonstrate that different plant families and species have specific secondary metabolites, and the same molecular elicitor can trigger diverse reactions and initiate broad changes in phytohormone levels (Iula et al., 2022).

Our examination shows that reactions to the administration of chitosan in the medium were rapid, observable within 7 days of culture, and gradually disappeared after 2 weeks. This study also shows a change in the profile of SL derivatives associated with a threefold higher content of helenalin esters compared to the control. In the Arbo variety, higher amounts of HT, HM, HIB, and HA were noted, while *A. montana* showed a slight increase in helenanolides, but a significant rise in dihydrohelenalin derivatives (DHM, DHT, and DHMB/DHIV).

Previous studies revealed that chitosan induces acquired resistance in plants to various pathogens by the production of proteins, enzymes, and secondary metabolites. It enhances the levels and activities of glucanase, chitinase, phenylalanine ammonia-lyase, polyphenol oxidase, superoxide dismutase, and catalase, promoting the synthesis of phytoalexins and the formation of suberin, lignin, and phenols (Hidangmayum et al., 2019).Chitosan itself also has a destructive effect on fungal and bacterial pathogens. In its low molecular mass water-soluble (LMWS) form, chitosan shows an affinity for plasmalemma lipids; its specificity in the microbial systems is associated with a positive charge, which promotes interaction with anionic components of the plasma membrane (Park et al., 2008). Using the technique developed by Park et al. (2008), which employs fluorescein isothiocyanate (FITC)-labeled chitosan derivatives (Geisberger et al., 2013), showed that labeled molecules attach to a microbial wall and intracellular ultrastructure in tested species. It seems interesting to consider whether the interaction with oil bodies inherent in cells capable of synthesizing phytoalexins triggers their biosynthesis.

JA exhibited effects similar to those of chitosan, but the enhancement of biosynthesis in the wild species was weaker, and the only effective dose was the lowest (0.2 mg/L). After applying both compounds, no significant changes in the SL profile were observed. However, the increase in SLs synthesis in the Arbo variety was greater than with chitosan, occurring across a wider range of doses.

JA is part of the signal transduction system that regulates the growth and response of plants to abiotic (cold, drought, salinity, metals, light) and biotic stresses. It operates within a complex signaling network involving phytohormones, integrating regulatory transcription factors and their associated genes, including those responsible for defense reactions. At a quantity of 10–^4^ M, it induces the synthesis of mimilactone A, the main phytoalexin in rice suspension culture (Nojiri et al., 1993). In-ground cultivation administering 1 mM methyl jasmonate (MeJA) with 0.025 mg/L of zinc increased menthol content in essential oil from *Mentha piperita*, affecting both its quality and quantity (Mehdizadeh et al., 2024). Wang et al. (2020) believe that JA does not play an independent role in the regulatory network, but acts by integrating with transcription factors of related genes while acting synergistically and antagonistically with abscisic acid, ethylene, and salicylic acid (Wang et al., 2020). The red light modulates, creating a synergistic regulatory effect on secondary metabolites, promoting JA accumulation or signaling. Pfr suppresses JAZ repressor, releasing MYC2, a key transcription factor for secondary metabolism. The red light also interacts with ABA and may enhance flavonoid accumulation during drought stress mediated by MYB/WRKY activation (Zvi et al., 2012; Johnson et al., 2023).

The receptor for JAs is the CORONATINE INSENSITIVE 1 (COI1) protein, which associates with Skp1-like, cullin 1, and ring-box protein to form the ubiquitin ligase SCF^COI1^ complex, participating in the degradation of negative regulators of JA signaling (Yan et al., 2009). The negative regulator of JA of the transcriptional network is the JASMONATE ZIM DOMAIN protein (JAZ). JAZ protein at the NT domain interacts with DELLA protein, inhibiting JA signaling. The C-terminus of the JAZ protein regulates function and binding to COI1 and MYC2/3/4 for transduction of downstream signaling. In the absence of JA, JAZ binds TF (MYC/MYB/WD40 and others), inhibiting genes that respond to JA. The presence of JA promotes recognition of JAZ by ubiquitin ligase. It directs this protein to degradation in the SCF^COI1^ complex, which releases TFs inhibiting or activating expression of JA-responsive genes (Zhao et al., 2024). In our experiments, the effect of the application of JA disappears after 10 days, which indicates rapid synthesis of new JAZ proteins after signal transduction (Zhao et al., 2024). Additionally, Yan et al. (2022) showed that *NbJAZ3* was highly expressed in trichomes, and lines with overexpression of *NbJAZ3* trichome density significantly decreased with lower levels of expression of *NbWo, NbCycB2, and NbMIXTA*. Lines of *NbJAZ3* RNAi slightly increased trichome density with higher expression of *NbWo*. The authors’ findings give a novel JA-mediated glandular trichome development model consisting of NbJAZ3-NbWo-NbCycB2 axis.

*Arnica montana* is protected in many countries, and for medicinal purposes, plant cultivation is introduced. Plantations of this species occupy a small area in Germany, France, and the Netherlands. In Poland, attempts were made to cultivate *Arnica montana*, but difficulties were encountered in establishing and maintaining cultivation (Kozłowski et al., 1999) (Sugier, 2007). achieved the first successes in the experiments by applying different elements of agricultural technology. On the investigational fields, the wild species grown from seedlings gave high yields of raw material in the third (97.5 g/m^2^) and fourth (99.7 g/m^2^) year of cultivation, while plants from shoot cuttings showed significantly lower harvests and the maximum flower yield was obtained earlier, in the third year of vegetation. Taking into account the variability of yield depending on the method of establishing the plantation in subsequent years, an average of 85.1 g of dry flower mass per square meter was annually obtained. The content of lactones in flowers ranges between 8–12 mg/g DW, which gives 600-1021.2 mg of lactones per m^2^ of cultivation. In the bioreactor, from the initial plant mass of 80 g FW, after 9 weeks, we obtain 13.82 g/g DW of SLs. The growing plant clump contains 84% leaves, with a weight of 32.78 g DW. The Arbo variety in the bioreactor contains, on average, 40.25 mg/g DW lactones, giving 1319.40 mg lactones at 100 µmol m^-2^ s^-1^ lighting, which is equivalent to the flower yield from approximately 1 m^2^ of ground cultivation. This quantity was obtained after one 9-week cycle in a bioreactor and not after several years of cultivation.

The content of lactones in leaves collected from a cold frame in the Botanical Garden in Wroclaw was estimated at 0.85 mg/g DW for the species and 8.40 mg/g DW for the variety (Parafiniuk et al., 2023). The species also contains significantly smaller amounts of lactones in the *in vitro* culture, which shifts into a lower reproduction coefficient. This feature is associated with faster maturation of rhizomes and roots, manifested by darkening and hardening of these tissues, which indicates a higher content of phenolic compounds. Analyzing the level of lactones in the wild species and the variety, it can be concluded that lactones are responsible for greater plant vigor. In the optimal environment of *in vitro* culture, this manifests itself in a reduced micropropagation rate, while in field conditions, it causes a low reproduction coefficient and a lack of plant expansion. In contrast, the variety quickly colonizes all available space. It can be argued that this is determined by the ratio of lactones to phenolic compounds in the internal tissues of both taxa. This places SLs in the category of compounds acting in a hormone-like manner, influencing cytokinin metabolism or influencing the GA and ABA ratio, which maintains significant plant juvenility and generates strong expansion in field cultivation.

The propagation of whole plants in the bioreactor is derived from observations of Malarz et al. (1993), who noted the influence of the root system on the level of SL biosynthesis and on our knowledge resulting from anatomical structure examination of the internal and external secretory system of *Arnica montana* (Kromer et al., 2016). The lipid distribution and fatty acid composition of triglycerides contained in the oil bodies of various organs were studied. The previously unknown connection of the axial internal secretory system of all organs into one coherent whole affects the availability of triglycerides contained in oil bodies as a substrate for the synthesis of essential oils, including SLs. In addition to the axial system of secretory channels, the plant has a system of discontinuous channels in the primary cortex and idioblasts that supply intermediates for the final synthesis of compounds stored in places critical for the health and ecological safety of the plant. For SLs, bitter substances, herbivore repellents, and perhaps also allelopathic compounds, the final site of biosynthesis is glandular hairs on the surface of leaves. The explanation presented above prompted us to use whole plant cultures in experiments with great success. The currently developed bioreactor cultivation method allows for a 7-fold increase in fresh mass increments within one month, which is serious competition in relation to extremely difficult field cultivation.

The extract from plants cultivated *in vitro* is devoid of even traces of pesticides, fungicides, or herbicides and is additionally sterile, which is currently required by the European Union. Production in bioreactors also does not pollute the environment, which is why, in our opinion, it is the best future solution. Our research can be applied to eliminate the harvesting of *Arnica montana*, a legally protected endangered plant, from its natural habitats, which also possesses significant beneficial medicinal properties. Therefore, production in bioreactors is the most suitable solution to protect endangered *A. montana* plants and produce sterile extracts for various applications.

Various techniques are employed to obtain secondary metabolites, utilizing methods such as cell suspension cultures, callus cultures, transformed organs, and whole plants. Typically, these cultures require the addition of phytohormones and involve several phases, including the selection of highly productive lines, the multiplication of plant material, and the production stage. The method we propose is relatively simple and cost-effective, not requiring hormone supplementation. Furthermore, when using tetraploid varieties of A. montana bred in the Plant Tissue Culture Laboratory and registered with COBORU (Research Center for Cultivar Testing), the efficiency of lactone biosynthesis can be significantly enhanced.

## 5 Conclusions

In chemical analysis of plant tissues, helenalin and dihydrohelenalin, along with their six corresponding esters with carboxylic acids, were identified.

Plants of *A. montana* and Arbo variety cultivated *in vitro* on MS medium form 3–7 adventitious shoots, with a cumulative weight increasing 5–7 times during 10 weeks. The Arbo variety had a higher reproduction rate; its shoots were juvenile, unlike those of the species that had earlier produced secondary growth. The primary site of SL biosynthesis is in leaves (40.26 mg/g DW), but shoots also contain significant amounts of these components (11.9-14.6 mg/g DW). The higher content of SLs suggests a defense and protective role for these compounds in young tissues under low light illumination, before substantial amounts of phenols are synthesized in sunlight, as well as their potential participation in promoting growth processes.

Both taxa examined in terms of the lactones represent separate types of plants that can be classified as subspecies. The Arbo variety contains significantly higher levels of SLs, with a more abundant helenalin fraction.

A chitosan concentration of 50 mg/L is effective for the *Arnica montana* species, while 100 mg/L is optimal for the Arbo variety. Chitosan enhanced the biosynthesis of the tested lactones, shifting the compound profile towards helenalin and its esters, including dihydrohelenalin and its derivatives.

Jasmonic acid, added to the MS medium as a regulator of responses to abiotic and biotic stresses, participates in the induction of defense reactions and initiates a 34% to 66% increase in the content of SL esters.

In both studied taxa, the equal influence of the photosynthetic photon flux density in the red, yellow, and blue ranges showed that only the light with the longest wavelength caused SLs accumulation with an increase in the biosynthesis of SLs, *A. montana* synthesized three times more lactones after 2 months of culture, while an increase of over 60% is detected in *Arnica montana* ‘Arbo’ compared to controls.

The biosynthesis of SLs in *in vitro* cultured plants of *A. montana* and the Arbo variety is regulated by light conditions. At a quantum flux density of 50 to 150 µmol m^-2^ s^-1^, the content of lactones increased by 36.6% in *A. montana* and 24.4% in the Arbo variety.

The high content of SLs in the cultured *in vitro* plant leaves of both species, especially at light intensity - 150 µmol m^-2^ s^-1^ (*A. montana* 32.57 mg/g DW; *A. montana* ‘Arbo’ 73.90 mg/g DW), creates the basis for the use of this method in the production of helenalin and dihydrohelenalin and their esters in bioreactors.

Future research should focus on integrating transcriptomic and metabolomic approaches to comprehensively elucidate the biosynthesis of SLs in *Arnica*. Such insights would deepen our understanding of the associated molecular interactions and facilitate subsequent genome editing using technologies like CRISPR/Cas9.

## Data Availability

The original contributions presented in the study are included in the article/supplementary material. Further inquiries can be directed to the corresponding authors.
